# MR-based calf muscle perfusion index correlates with treadmill exercise test parameters in patients with peripheral arterial disease

**DOI:** 10.1186/1532-429X-15-S1-O58

**Published:** 2013-01-30

**Authors:** Stephanie Clement-Guinaudeau, Matthew L Topel, Arshad Ali, Joseph C Poole, Elizabeth Rocco, Shafaat A Khan, Xiaodong Zhong, Frederick H Epstein, Christopher M Kramer, Arshed A Quyyumi, John N Oshinski

**Affiliations:** 1 Emory University, Atlanta, GA, USA; 2 Georgia Institute of Technology, Atlanta, GA, USA; 3 University of Virginia, Charlottesville, VA, USA; 4 Siemens Healthcare, Atlanta, GA, USA

## Background

The functional impairment caused by peripheral arterial disease (PAD) is difficult to evaluate objectively and quantitatively. Current methods used to assess the efficacy of therapeutic interventions in patients with PAD are limited by variability and changes representing the placebo effect. Recently, Gadolinium-enhanced first-pass (FP) MRI has emerged as a new method to assess perfusion in peripheral muscles at peak exercise.

We seek to demonstrate that calf muscle perfusion index measured with FP MRI at peak exercise correlates with treadmill exercise measures of ischemia in patients with PAD.

## Methods

**Figure 1:**
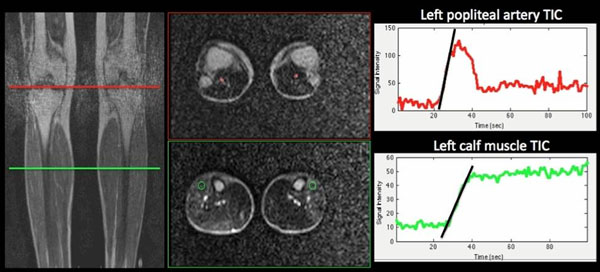
Analysis of FP MRI images

82 patients with PAD were included in the study. Patients exercised until fatigue inside the 1.5T MR scan using an MR-compatible plantar-flexion ergometer. Images were acquired at peak exercise using a dynamic, first-pass, dual-contrast sequence. The dual contrast sequence acquires two interleaved slices; an inversion-prepared slice in the calf muscle, and a saturation-prepared slice in the popliteal artery (8 cm superior). Time-intensity curves (TIC) were acquired from the dynamic images by placing ROI's in the popliteal artery on the saturation-prepared slices in the brightest calf muscle group on the inversion-prepared slice (Figure 1). The perfusion index (PI) = calf muscle TIC upslope / popliteal artery TIC upslope. Graded treadmill exercise testing was performed using the Gardner protocol. Claudication onset time (COT) and total walking time (TWT) were recorded for each patient, as well as resting ABI.

## Results

62 patients were able to complete both the MRI and treadmill test. The PI measured by MRI correlated significantly with both times measured during the treadmill exercise test, the TWT and the COT (r =0.33 p<0.01, r=0.29 p=0.01, respectively). The PI was not correlated with resting ABI.

## Conclusions

MR-based calf muscle PI correlates significantly with treadmill exercise parameters. MRI PI could be a valuable tool to objectively and quantitatively assess improvement in exercise perfusion in response to therapeutic intervention in patients with PAD.

## Funding

Funding was provided by a grant from the National Institutes of Health (RC2HL101515 to Arshed Quyyumi, MD).

